# Phytochemical Variations in Spiny and Spineless Safflower (*Carthamus tinctorius* L.) Phenotypes

**DOI:** 10.3390/plants15192959

**Published:** 2026-09-28

**Authors:** Cătălina Tudora, Adriana-Teodora Muscalu, Valentin-Nicolae Vlăduț, Ana-Cristina Fătu, Danela Murariu, Florentina Gatea

**Affiliations:** 1 Testing Department, National Institute of Research-Development for Machines and Installations Designed for Agriculture and Food Industry-INMA, 6, Ion Ionescu de la Brad Avenue, 013813 Bucharest, Romania; amuscalis@yahoo.com (A.-T.M.); valentin_vladut@yahoo.com (V.-N.V.); 2 Research-Development Institute for Plant Protection-ICDPP Bucharest, 8, Ion Ionescu de la Brad Blvd, District 1, 013813 Bucharest, Romania; catos2411@yahoo.com; 3 Banca de Resurse Genetice Vegetale “Mihai Cristea”, 17, 1 Mai Avenue, 720224 Suceava, Romania; danela.murariu@svgenebank.ro

**Keywords:** *Carthamus tinctorius* L., underutilized crops, HPLC-DAD, polyphenolic profile, fat-soluble vitamins, natural dyes

## Abstract

Currently, documentation in the scientific literature regarding the specific phytochemical variations among regionally adapted spiny and spineless safflower (*Carthamus tinctorius* L.) germplasm remains limited. This study evaluated the biochemical profiles of petals from four Romanian local populations representing spiny (var. *typicus*) and spineless (var. *inermis*) phenotypes. Screening via spectrophotometric methods and HPLC-DAD targeted total phenolic content (TPC), total flavonoid content (TFC), individual phenolic compounds, fat-soluble vitamins, total dye yield, and antioxidant capacity (DPPH, ABTS, FRAP). The spineless population R4-CL recorded the highest levels of TPC (25.26 ± 0.29 mg GAE g^−1^ d.w.), natural dye yield (5.56%), and vitamin E (8.45 ± 0.19 mg 100 g^−1^ d.w.) using 100% ethanol extraction. Chlorogenic and caffeic acids emerged as the primary phenolic acids, whereas vitamin K_1_ was not detected. Spiny genotypes exhibited superior DPPH scavenging efficiency, whereas FRAP values remained statistically homogeneous. These findings demonstrate that the evaluated spineless local lines possess promising phytochemical profiles while offering agronomic benefits for handling. Although limited to a single growing season and a single location, this study provides useful preliminary baseline data on the localized chemical traits of these Romanian landraces.

## 1. Introduction

Genetic and functional diversity in horticultural crops is essential for ensuring food security and enhancing agroecosystem resilience against climate change, diseases, and pests. According to the Food and Agriculture Organization (FAO), a significant proportion of global agricultural genetic diversity has been compromised over the past century due to the homogenization of agronomic practices [1]. To safeguard biodiversity and promote sustainable development, researchers are increasingly shifting their attention toward neglected and underutilized horticultural species (NUS) that exhibit superior bioactive and nutritional profiles compared to widely cultivated commercial monocultures [1,2]. Due to their robust adaptation to harsh agroecological conditions, NUS offer the opportunity to expand cultivation onto marginal lands, thereby supporting local economic development, food security, and organic horticultural systems [1,2].

Safflower (*Carthamus tinctorius* L., Asteraceae) is a classic example of an underutilized crop with multiple values in the medicinal and niche horticultural plant sectors. Traditionally cultivated for its flowers to obtain natural dyes and food flavorings, this crop experienced a sharp decline with the advent of synthetic aniline dyes [2]. Safflower petals are rich in water-soluble yellow pigments (carthamidin and safflomin fractions) and hydrophobic red pigments (carthamin), providing non-toxic natural colorants highly demanded in the food, textile, pharmaceutical, and cosmetic industries. However, the commercial expansion of this crop for exclusive petal exploitation is restricted by the presence of spines (var. *typicus*), which renders manual harvesting a labor-intensive and costly operation [3,4]. In this regard, the characterization of spineless genotypes (var. *inermis*) represents a priority direction in horticultural research, facilitating mechanization without compromising phytochemical quality [4]. Concurrently, evaluating the phenotypic plasticity and regional adaptability of these spineless cultivars remains a major focus in modern germplasm screening, as local climate and soil interactions significantly shape both biomass yields and secondary metabolite accumulation [5]. Understanding these regional agronomic responses within wider historic breeding frameworks is crucial for expanding safflower as a resilient option under shifting environmental stresses [6]. Although it is one of mankind’s oldest crops, historically grown on small plots for domestic use, safflower remains a minor crop globally, with a world seed production of approximately 800,000 tons per year [2,3,4,7,8]. Characterizing and conserving these genetic resources are vital for sustainable horticulture and functional food design [8]. In the 1990s, Italy intensively evaluated safflower under the “Oilseed Seeds” project, demonstrating viable productivity and premium quality of biomass on marginal lands in central-southern regions [9,10,11,12,13]. Similarly, India remains a major producer, where interest in safflower petals has experienced a massive resurgence due to safety concerns associated with synthetic azo dyes [14].

In Romania, agronomic interest in safflower experienced an expansion phase two decades ago, with the cultivated area growing significantly [15]. The plant is of particular importance for drought-prone zones and low fertility soils, where it ensures yields comparable to traditional crops. However, the successful introduction of safflower into current Romanian horticultural systems requires a preliminary evaluation of its germplasm to verify its adaptability to contemporary climatic conditions. Although previous studies have isolated various phenolic compounds from safflower, detailed comparative data regarding the variation in these biochemical profiles and associated antioxidant capacities as a function of local morphological varieties remain extremely limited [16,17]. Furthermore, while most studies focus exclusively on hydrophilic pigments, the potential of safflower petals as a source of essential fat-soluble lipophilic vitamins, primarily vitamin E (α-tocopherol), remains relatively unexplored within local germplasm, despite the rising demand for natural bioactive ingredients in the nutraceutical industry [17,18].

To address this literature gap, the primary objective of this work was to provide a preliminary biochemical characterization of petals harvested from local Romanian safflower germplasm, comparing two distinct morphological types: spiny populations (var. *typicus*, originating from the Moldavia region) and spineless populations (var. *inermis*, originating from the Muntenian region). By evaluating total phenolics, total flavonoids, specific fat-soluble vitamin fractions (Vitamin E and K_1_), total dye content, and antioxidant capacity via HPLC-DAD, this study aims to establish a localized baseline regarding the potential of these local safflower lines, providing foundational data for the future integration of underutilized genotypes into sustainable horticultural frameworks.

## 2. Materials and Methods

### 2.1. Plant Materials and Growth Conditions

The biological material used in this study consisted of the dried petals of four local populations and genotypes of safflower (*Carthamus tinctorius* L., Asteraceae). The original seeds were obtained from the “Mihai Cristea” Plant Genetic Resources Bank in Suceava, Romania, where the respective parental germplasm is officially registered and maintained. The evaluated germplasm belongs to two distinct morphological varieties: spiny genotypes (*C. tinctorius* L. var. *typicus*) and spineless genotypes (*C. tinctorius* L. var. *inermis*). Field trials tracked the chronological development of the crop across major ontogenetic stages to document phenotypic adaptation under regional climatic conditions (Figure 1a–d).

- **Spiny genotypes** (*C. tinctorius* L. var. *typicus*): (i) Population 1: Local population Botoșani, Dorohoi location, coded **R1-BT** (Figure 1e; Accession No.: SVGB-4632); (ii) Population 2: Local population Bacău, Oncești location, coded **R2-BC** (Figure 1f; Accession No.: SVGB^−1^1778).
- **Spineless genotypes** (*C. tinctorius* L. var. *inermis*): (iii) Population 3: Local population Teleorman, Teleormanu location, coded **R3-TR** (Figure 1g; Accession No.: SVGB-4621); (iv) Population 4: Genotype/Line Călărași, obtained from INCDA Fundulea, coded **R4-CL** (Figure 1h; Accession No.: SVGB^−1^0326).

**Figure 1:**
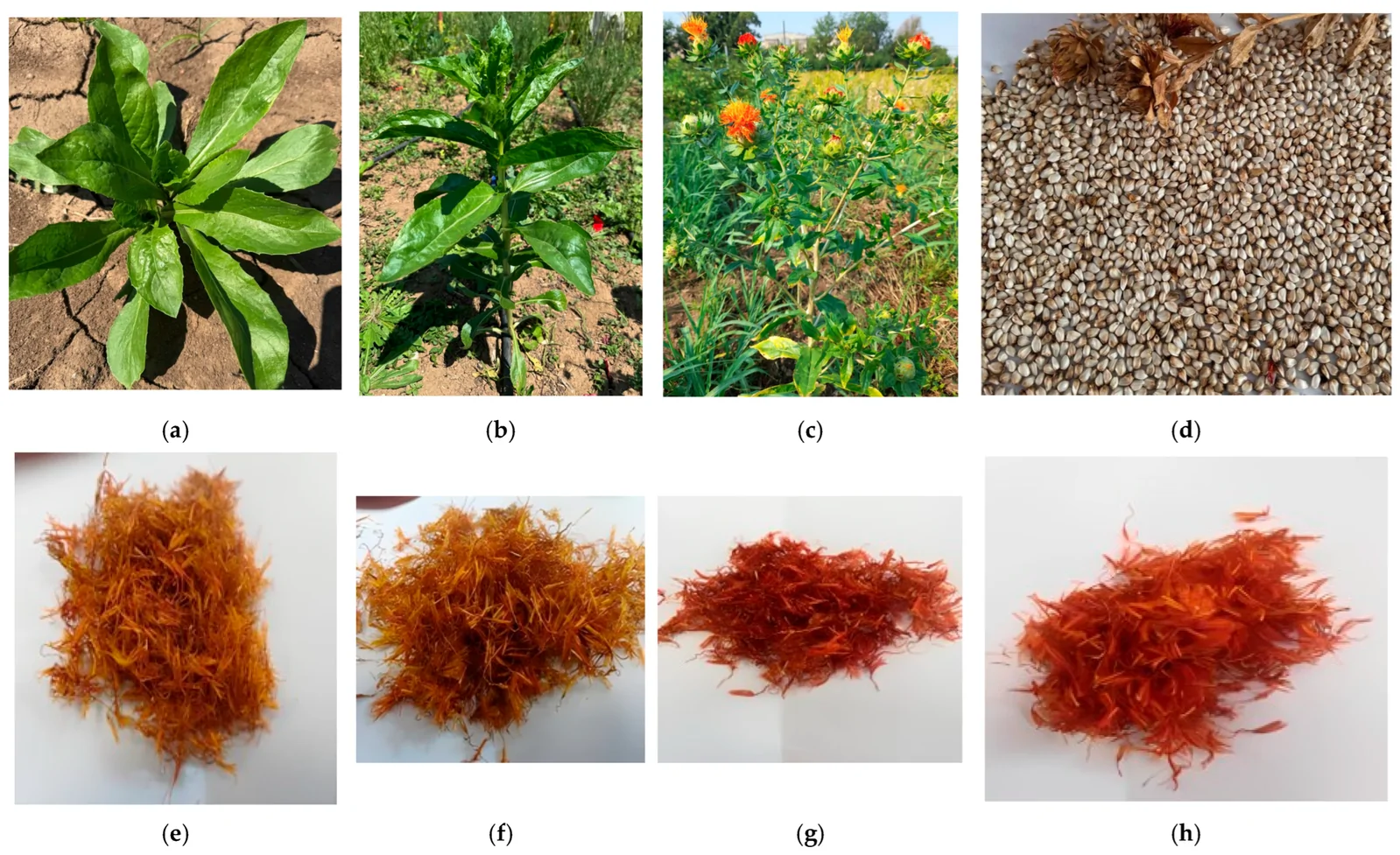
Chronological development and morphological documentation of *Cantharus tinctorial* L. during the 2024 cultivation cycle: (**a**) early vegetative establishment (rosette phase); (**b**) vegetative growth phase; (**c**) field overview during full anthesis (peak flowering stage); (**d**) crop at physiological maturity; (**e**–**h**) evaluated biological material consisting of dried safflower petals from local populations and genotypes: (**e**) R1-BT, (**f**) R2-BC, (**g**) R3-TR, and (**h**) R4-CL.

The field experiment was conducted during the 2024 vegetative season (Figure 1a–d). Due to the limited initial seed stock available from the “Mihai Cristea” Plant Genetic Resources Bank (Suceava), the scope of this primary cultivation cycle was focused exclusively on the baseline phytochemical characterization of the petals (Figure 1e–h). While a single-season evaluation cannot capture long-term climate-induced variations, this approach was designed as a preliminary germplasm screening to assess the immediate chemotypic differentiation between spiny and spineless phenotypes under identical environmental and agrotechnical conditions. Consequently, these findings establish a necessary metabolic baseline, providing the justification for future multi-year and multi-location trials as germplasm availability expands.

To obtain the experimental petal samples, a field trial was established in the Bucharest-Banes experimental area (lat. 44°30′01″ N, long. 26°04′19″ E, alt. 90 m above sea level) under a temperate-continental climate (Figure 2a). The regional, national, and high-resolution satellite layout of the cultivation plots at the National Research-Development Institute for Machines and Installations Destined to Agriculture and Food Industry (INMA Bucharest) are anchored to clarify the geographic localization of the evaluated landraces (Figure 2b). The crop was cultivated on a soil type classified as a Pelvic Lucidol according to the World Reference Base (WRB) for Soil Resources.

Sowing was performed in early April 2024. The total experimental area covered 50 m^2^, following standard agronomic practices for safflower cultivation, without the application of artificial irrigation or synthetic chemical treatments. Harvesting of the petals was performed manually at the full flowering stage (complete anthesis) of the capitula in July–August 2024. The collected petals were air-dried in the shade at room temperature (22 ± 2 °C) until a constant weight was achieved. The dried samples were stored in opaque paper bags under dark conditions to prevent the photochemical degradation of bioactive compounds. Prior to biochemical extraction, the petals were homogenized into a fine powder using a laboratory porcelain mortar. Crude extracts were subsequently prepared according to the specific analytical protocols detailed in the following subsections. Since only two distinct populations were evaluated per morphological variety, the observed trends between the spiny and spineless lines are interpreted strictly as phenotypic observations descriptive of these specific local genotypes, rather than generalized or definitive differences between the broader morphological varieties (var. *typic us* and var. *interims*).

### 2.2. Preparation of Methanolic Extracts

For the determination of total phenolic content, total flavonoid content, antioxidant capacities, and individual phenolic compounds, a common ultrasound-assisted extraction (UAE) protocol was applied. Briefly, 0.1 g of dried petal powder was extracted with 5 mL of 80% (*v*/*v*) aqueous methanol. The extraction process was performed in an ultrasonic bath Elmasonic P (Elma Schmidbauer GmbH, Singen, Germany) operating at a fixed frequency of 37 kHz, an ultrasonic power of 100 W, and a controlled temperature of 50 °C for 1 h. The solid-to-solvent ratio was fixed at 0.1:5 (g mL^−1^). Following the extraction process, the mixture was centrifuged at 5000 rpm for 10 min to separate and collect the clear supernatant used for the subsequent assays (crude extract).

### 2.3. Determination of Total Phenolic Content (TPC) and Total Flavonoid Content (TFC)

The quantification of total polyphenols was performed spectrophotometrically using the colorimetric Folin–Ciocalteu method [19]. Briefly, the diluted crude methanolic extract (2 mg mL^−1^) (obtained as described in Section 2.2) was thoroughly mixed with the Folin–Ciocalteu reagent, followed by the addition of a 7.5% (*w*/*v*) aqueous sodium carbonate (Na_2_CO_3_) solution and distilled water. The mixture was subsequently incubated in the dark at 50 °C for 1 h. After incubation, the absorbance was measured at a wavelength of 760 nm against a blank solution containing 80% methanol. The total phenolic content was calculated based on a gallic acid calibration curve prepared from a 1 mg mL^−1^ stock solution (*R*^2^ ≥ 0.99), and the final quantitative results were expressed as milligrams of gallic acid equivalents per gram of dry weight (mg GAE g^−1^ d.w.).

Concurrently, the total flavonoid content (TFC) in the same methanolic extracts was determined according to the aluminum chloride colorimetric method. Briefly, the diluted crude sample extract (6 mg mL^−1^) (prepared as detailed in Section 2.2) was thoroughly mixed with a 10% (*w*/*v*) AlCl_3_ solution and a 1 M sodium acetate (CH_3_COONa) solution. Following an incubation period at room temperature for 60 min, the absorbance of the reaction mixture was measured at 415 nm. The quantification of total flavonoids was performed based on a quercetin calibration curve prepared from a 1 mg mL^−1^ stock solution (*R*^2^ ≥ 0.99), and the results were expressed as milligrams of quercetin equivalents per gram of dry weight (mg QE g^−1^ d.w.).

### 2.4. Evaluation of Antioxidant Capacity

The antioxidant capacity of the safflower petal extracts was evaluated using three distinct complementary assays using the crude methanolic extracts prepared according to Section 2.2.

#### 2.4.1. DPPH Assay (mM Trolox and IC_50_ Calculation)

The 2,2-diphenyl-1-picrylhydrazyl (DPPH) free radical scavenging activity was determined according to the method reported by Brand-Williams et al. [20]. Briefly, the diluted crude sample extract (0.3, 0.5, 1, 1.3 and 1.5 mg mL^−1^) was mixed with a freshly prepared methanolic solution of DPPH (0.25 mM). The diluted sample extract was mixed with a freshly prepared methanolic solution of DPPH. The mixture was incubated in the dark at room temperature for 30 min, and the decrease in absorbance was monitored at 517 nm against a methanol blank. The percentage of DPPH radical scavenging activity was calculated using the following equation:

DPPH Scavenging (%) = [(A_blank_ − A_sample_)/A_blank_] × 100 (1)

where A_blank_ represents the absorbance of the DPPH solution without the sample, and A_sample_ represents the absorbance of the reaction mixture containing the extract. The antioxidant efficiency was calculated based on a Trolox calibration curve (*R*^2^ ≥ 0.99) and expressed as milligrams of Trolox equivalents per gram of dry weight (mg TE g^−1^ d.w.), as well as evaluated via the semi-maximal inhibitory concentration (IC_50_). Based on literature standards, the antioxidant potential was classified by IC_50_ values as follows: strongly active (IC_50_ < 50–100 µg mL^−1^), moderately active (101–250 µg mL^−1^), and weakly active or inactive (>250 µg mL^−1^).

#### 2.4.2. ABTS Assay (mM Trolox)

The Trolox Equivalent Antioxidant Capacity (TEAC) was evaluated via the 2,2′-azino-bis (3-ethylbenzothiazoline-6-sulfonic acid) (ABTS^+^) radical cation decolorization assay, as described by Re et al. [21]. The ABTS^+^ radical cation was generated by reacting the ABTS stock solution (7 mM) with potassium persulfate (2.5 mM, K_2_S_2_O_8_) in the dark at room temperature for 12–16 h before use. The solution was subsequently diluted with methanol to achieve an initial absorbance of approximately 0.7000 ± 0.020 at 734 nm. For the analysis, the diluted crude floral extract (0.6 mg mL^−1^) was mixed with the diluted ABTS^+^ solution. After the designated incubation period, the absorbance was measured at 734 nm. The scavenging activity was quantified using a Trolox calibration curve (*R*^2^ ≥ 0.99), and the results were expressed as millimoles of Trolox equivalents per gram of sample (mM Trolox g^−1^ d.w.).

#### 2.4.3. FRAP Assay (mM Trolox)

The ferric reducing capacity was determined in accordance with the modified colorimetric protocol described by Benzie and Strain [22]. The working FRAP reagent was prepared fresh by mixing 10 volumes of 300 mM acetate buffer (pH 3.6), 1 volume of 2,4,6-tripyridyl-s-triazine (TPTZ) dissolved in 40 mM HCl, and 1 volume of 20 mM FeCl_3_. Subsequently, 3000 µL of the FRAP reagent (pre-heated to 37 °C) was vigorously mixed with 100 µL of the methanolic safflower extract (0.6 mg mL^−1^). The reaction was carried out at 37 °C for 4 min, after which the absorbance was measured spectrophotometrically at 593 nm against a reagent blank. Calibration curves were constructed using iron sulfate (FeSO_4_) and Trolox, both prepared from a 1 mM stock solution. The final FRAP values were expressed as millimoles of FeSO_4_ equivalents per gram (mM FeSO_4_ g^−1^) and millimoles of Trolox equivalents per gram (mM Trolox g^−1^) of dry petal weight.

All spectrophotometric measurements were performed using a Jasco V-630 UV/VIS spectrophotometer (Jasco, Tokyo, Japan).

### 2.5. HPLC-DAD Profiling of Individual Phenolic Compounds

The individual polyphenolic compounds quantified in this study included gallic acid, protocatechuic acid, catechin, chlorogenic acid, caffeic acid, *p*-coumaric acid, rutin, ferulic acid, isoquercetin, rosmarinic acid, myricetin, quercetin, apigenin, naringenin, kaempferol, and isorhamnetin. This panel of reference standards was proactively selected based on global phytochemical literature for the genus *Carthamus* to perform an extensive screening and avoid false-negative omissions. The fact that some compounds remained below the limits of detection (LOD) represents a finding that defines the phytochemical fingerprint of these local Romanian landraces compared to international varieties. The concentration of each analyte was expressed as milligrams per 100 g of dry weight (mg 100 g^−1^ d.w.).

The crude methanolic extracts prepared according to Section 2.2 were directly used for High-Performance Liquid Chromatography coupled with a Diode Array Detector (HPLC-DAD) (Shimadzu Corporation, Kyoto, Japan) profiling. The analytical system consisted of a Shimadzu LC-20A Prominence equipment (Shimadzu Corporation, Kyoto, Japan) equipped with an LC-20AT quaternary pump and a diode array detector, utilizing an internally validated in-house method (Supplementary Materials Table S2). Instrument calibration was performed using high-purity chromatographic standards (>95%, Sigma-Aldrich, St. Louis, MO, USA). Chromatographic separation was carried out on a Kinetex C18 reversed-phase column (150 × 4.6 mm, 5 µm particle size, Phenomenex, Torrance, CA, USA) maintained at 35 °C and the injection volume was 10 µL. The mobile phase was delivered in a gradient mode at a constant flow rate of 0.8 mL min^−1^, consisting of water acidified with phosphoric acid, pH 2.3 (Mobile Phase A) and acetonitrile (Mobile Phase B). Before analysis, the mobile phase was filtered through LLG syringe filters (PVDF, 0.2 µm, MACHEREY-NAGEL GmbH & Co. KG (Düren, Germany) and degassed. The working gradient for polyphenols separation was: 0–25 min, 5–50% B; 25–35 min, 50–65% B; 35–39 min, 65–30% B; 39–41 min, 30–5% B; 41–45 min, 5%. The monitoring of the target analytes was executed at specific wavelengths of 280 (gallic acid, protocatechuic acid, catechin, cinnamic acid, apigenin), 320 (caffeic acid, chlorogenic acid, rosmarinic acid, *p*-coumaric acid, ferulic acid, naringenin), and 360 nm (rutin, kaempherol, quercetin, isoquercetin), depending on the maximum UV-Vis absorption spectrum characteristic of each compound. The identification of individual compounds was based on the comparison of retention times and the verification of chromatographic peak purity against reference standards. Quantification was determined using external standard calibration curves (*R*^2^ ≥ 0.99). Data acquisition, peak integration, and result processing were executed using the Shimadzu LC-solution software (Version 5.51).

### 2.6. Quantitative Analysis of Fat-Soluble Vitamins

The fat-soluble vitamins targeted for identification and quantification in the safflower petal samples included vitamin E (α-tocopherol) and vitamin K_1_ (phylloquinone), from Sigma-Aldrich, Merck (Darmstadt, Germany). The concentration of each vitamin was expressed as milligrams per 100 g of dry weight (mg 100 g^−1^ d.w.).

The extraction of fat-soluble vitamins from the dried petals was performed using two distinct solvent systems: (a) a polar solvent consisting of 100% ethanol, and (b) a non-polar solvent mixture consisting of hexane:ethyl acetate (4:1, *v*/*v*). For each procedure, a 0.2 g aliquot of dried petal powder was mixed with 30 mL of the respective extraction solvent. The mixtures were subjected to extraction in an ultrasound-assisted extraction bath operating at a frequency of 37 kHz, an ultrasonic power of 100 W, and a controlled temperature of 30 °C for 30 min. Following sonication, the samples were centrifuged at 5000 rpm to separate the supernatant, which was subsequently evaporated to dryness under vacuum. The resulting dry residue was reconstituted in 2 mL of HPLC-grade methanol.

The quantification of the target analytes was conducted using an in-house validated protocol (Supplementary Materials) adapted from Saini and Keum operating as a screening and comparative procedure optimized for baseline plant evaluation. Analytical separation and quantification were achieved using a Shimadzu LC-20A Prominence HPLC system (Shimadzu Corporation, Kyoto, Japan) equipped with an LC-20AT quaternary pump and a diode array detector (DAD). Chromatographic separation was performed on a Kromasil C18 reversed-phase column (150 × 4.6 mm, 5 µm particle size; AkzoNobel, Bohus, Sweden) operated in isocratic mode. The mobile phase consisted of a mixture of acetonitrile (Mobile Phase A) and methanol (Mobile Phase B) (50:50, *v*/*v*), delivered at a constant flow rate of 0.8 mL min^−1^. The column oven temperature was maintained at 30 °C and injection volume was 10 µL. The monitoring and quantitative integration of the target analytical peaks via DAD were executed setting the maximum absorption wavelengths (λmax) at 292 nm for vitamin E and 248 nm for vitamin K1. Quantification was achieved based on external calibration curves constructed using high-purity standard compounds (R^2^ ≥ 0.99) (Supplementary Materials Table S2). Data acquisition, peak integration, and result processing were executed using the Shimadzu LC-solution software. LOD and LOQ values were determined by regression analysis of the calibration curve (LOD = 3.3 × standard error/slope and LOQ = 10 × standard error/slope).

### 2.7. Determination of Total Dye Content

The extraction and yield determination of total natural dyes from safflower petals were performed via acid hydrolysis, following the protocol described by Erbaş and Mutlucan [23] with minor modifications. Briefly, a 0.3 g aliquot of dried plant material was subjected to acid hydrolysis using 2 M hydrochloric acid (HCl) for 30 min at 100 °C to release the bound pigment compounds. Upon completion of the hydrolysis process, the resulting colored extracts were filtered, washed thoroughly with distilled water until neutrality to remove residual acid, and subsequently evaporated to dryness under vacuum using a vacuum drying oven. The total dye content yield was determined gravimetrically and expressed as a percentage (%) relative to the initial dry weight of the plant material.

### 2.8. Statistical Analysis

All experimental determinations were conducted in three independent analytical replicates processed from a single homogeneous composite average sample per genotype, and the results are expressed as mean ± standard deviation (SD). The statistical significance and variations among the four local safflower landraces (R1-BT, R2-BC, R3-TR, and R4-CL) were evaluated using one-way Analysis of Variance (one-way ANOVA). To identify significant differences between the means, Tukey’s Honestly Significant Difference (HSD) post-hoc test was applied. The threshold for statistical significance was set at *p* < 0.05. All statistical calculations and data processing were executed using Microsoft Excel 365 and IBM SPSS Statistics software (Version 26.0; IBM Corp., Armonk, NY, USA). It is acknowledged as a limitation that the three analytical replicates were derived from a single homogeneous composite batch per genotype to reflect processing uniformity and instrument precision, rather than independent biological field replicates. Consequently, the resulting ANOVA *p*-values and Tukey’s post-hoc parameters describe the significance of variations between these specific composite profiles under our controlled laboratory conditions, serving as a preliminary baseline characterization.

## 3. Results

### 3.1. Total Phenolic Content (TPC) and Total Flavonoid Content (TFC) of Safflower Petal

The screening of the total phenolic content (TPC) and total flavonoid content (TFC) across the four local Romanian safflower landraces revealed considerable amounts of bioactive secondary metabolites, with distinct profiles observed among the tested genotypes. As is presented in Table 1, the investigated local germplasm exhibited TPC values within a narrow but statistically significant range, varying from 23.72 ± 0.13 mg GAE g^−1^ d.w. in the spiny population from Bacău (R2-BC) to a maximum of 25.26 ± 0.29 mg GAE g^−1^ d.w. in the spineless population from Călărași (R4-CL). An interesting aspect highlighted by the evaluation of general morphological traits was the observed tendency within these specific evaluated genotypes (var. *inermis*: R3-TR and R4-CL) to accumulate higher amounts of total polyphenols compared to the investigated spiny lines (var. *typicus*: R1-BT and R2-BC). According to the statistical analysis, the difference between the two spineless populations (R3-TR and R4-CL) was not statistically significant (*p* > 0.05), with both assigned to the same superior group (a). Conversely, the spiny populations recorded significantly lower concentrations and were separated into distinct statistical groups: R1-BT in group (b) and R2-BC in group (c).

Regarding the total flavonoid content (TFC), the results are consolidated alongside TPC in Table 1, expressed as milligrams of quercetin equivalents per gram of dry weight (mg QE g^−1^ d.w.). The values ranged from 8.18 ± 0.13 mg QE g^−1^ d.w. in the R2-BC population to 8.81 ± 0.26 mg QE g^−1^ d.w. in the R3-TR population. In contrast to the total phenolics, no statistically significant differences were observed among the experimental populations based on Tukey’s HSD test (*p* > 0.05), indicating a homogeneous distribution of this specific metabolic fraction across both spiny (var. *typicus*) and spineless (var. *inermis*) Romanian genotypes. These results underscore that Romanian local landraces represent a rich source of polyphenols, providing essential baseline biochemical data that demonstrate their potential for applications in the functional food or pharmaceutical industries within the current context of climate change.

### 3.2. Antioxidant Capacity of Safflower Petals Extracts

The evaluation of the antioxidant capacity of plant extracts requires complementary multi-method approaches to account for the diverse chemical structures and reaction mechanisms involved in free radical scavenging and ferric reducing power. The antioxidant profiles of the four Romanian safflower landraces, determined via DPPH, ABTS, and FRAP assays, are summarized in Table 2.

The experimental results revealed assay-specific variations regarding the antioxidant potential of the studied landraces. In the DPPH assay, the spiny genotypes (var. *typicus*) exhibited a superior radical scavenging efficiency, recording the lowest semi-maximal inhibitory concentration values. The landrace from Bacău (R2-BC) displayed the most pronounced antioxidant effect (IC_50_ = 12.09 ± 0.12 µg mL^−1^), followed closely by the Botoșani population (R1-BT, 12.45 ± 0.01 µg mL^−1^), both being assigned to the same statistical group (b) and being significantly more active (*p* < 0.05) than the spineless variants. Conversely, the spineless populations (var. *inermis*) showed higher IC_50_ values, namely 14.12 ± 0.00 µg mL^−1^ for R4-CL and 14.70 ± 0.28 µg mL^−1^) for R3-TR. A different trend was documented for the ABTS assay, where the spineless landrace from Călărași (R4-CL) recorded the highest radical cation scavenging capacity (145.71 ± 2.21 mM Trolox g^−1^), placing it in the superior homogenous group (a). The other three populations exhibited lower values, ranging from 137.28 ± 2.82 mM Trolox g^−1^ (R3-TR) to 140.14 ± 4.06 mM Trolox g^−1^ (R1-BT). Regarding the ferric reducing antioxidant power (FRAP assay), statistical analysis revealed the absence of any statistically significant differences (*p* > 0.05) among all four tested genotypes, showing homogeneous values for both FeSO4 equivalents (113.28 ± 5.06 mM FeSO_4_ g^−1^) and Trolox equivalents (118.01 ± 2.47 mM Trolox g^−1^).

### 3.3. Quantitative Analysis of Individual Phenolic Compounds

To elucidate the specific molecular composition of the extracts, a qualitative and quantitative evaluation was performed via HPLC-DAD. It must be specified that these chromatographic identifications remain provisional, HPLC-DAD screening cannot fully rule out co-elution events within the complex matrix of safflower petals. Among the profiled analytes, major and well-resolved constituents like chlorogenic acid, caffeic acid, and rutin are considered the most reliable due to their intense, symmetry-driven chromatographic signals and stable baseline resolutions. Conversely, trace constituents or those eluting in crowded regions, such as gallic acid and apigenin, are considered the least reliable and must be treated with caution, as they are highly susceptible to spectral overlaps or co-elution with matrix-specific quinochalcone isomers. Consequently, the reported quantitative mean and standard deviation values reflect the analytical reproducibility of the reference standard equivalents under the tested conditions, rather than absolute molecular confirmation. The concentrations of individual phenolic acids and flavonoids across the four Romanian safflower landraces, expressed as milligrams per 100 g of dry weight (mg 100 g^−1^ d.w.), are detailed in Table 3.

The chromatographic profiling revealed quantitative variations in the phytochemical fingerprint among the studied local landraces. Phenolic acids were primarily represented by hydroxycinnamic acid derivatives, among which chlorogenic acid emerged as the primary chromatographic peak monitored visually, whereas caffeic acid was determined as the primary constituent from a quantitative standpoint across all samples. The spineless landraces accumulated notable concentrations of caffeic acid, reaching a maximum value of 289.41 ± 5.85 mg 100 g^−1^ d.w. in R4-CL, followed by R3-TR (260.64 ± 4.11 mg 100 g^−1^ d.w.). Chlorogenic, *p*-coumaric, and ferulic acids were quantified across all lines. The R2-BC population recorded the highest concentration of chlorogenic acid (45.71 ± 1.13 mg 100 g^−1^ d.w.) and *p*-coumaric acid (57.70 ± 1.54 mg 100 g^−1^ d.w.). Gallic acid was detected in two lines, being absent in R2-BC. Although the R4-CL phenotype showed a clear and high peak for gallic acid, the standard deviation was extremely high. Therefore, this result was reported as not quantifiable (NQ), likely due to matrix interference or co-elution with another compound. Specifically, the high standard deviation noted for gallic acid in the R4-CL population reflected a localized baseline interference, leading to insufficient reproducibility of the chromatographic integration. Consequently, to prevent data over-interpretation, this analyte was conservatively designated as non-quantifiable (NQ), highlighting the necessity for advanced mass spectrometry profiling or further chromatographic optimization in subsequent validation trials.

Regarding the flavonoid fraction, the glycosylated flavonols (rutin and isoquercetin) were identified in all samples, with the maximum values recorded in the spiny line R1-BT (95.47 ± 8.69 mg 100 g^−1^ d.w. and 72.50 ± 4.61 mg 100 g^−1^ d.w. respectively). The differences in rutin content were not statistically significant among the populations (*p* > 0.05). Free aglycones, namely quercetin and isorhamnetin, were exclusively detected in sample R1-BT. Conversely, apigenin and naringenin recorded the highest levels in the spineless lines, reaching maximum concentrations in sample R4-CL (26.30 ± 0.25 mg 100 g^−1^ d.w. and 35.76 ± 0.29 mg 100 g^−1^ d.w. respectively), with statistically significant differences (*p* < 0.05). The kaempferol content was significantly higher in the spineless lines R3-TR (22.29 ± 0.36 mg 100 g^−1^ d.w.) and R4-CL (20.04 ± 0.30 mg 100 g^−1^ d.w.) compared to the spiny line R1-BT (8.14 ± 0.22 mg 100 g^−1^ d.w.), dropping below the detection limit in sample R2-BC. This distinct metabolic compartmentalization highlights that while spineless lines favor the biosynthetic pathways of flavones and specific flavonols, the spiny lines conserve broader pathways of free aglycones, providing valuable markers for the breeding and industrial exploitation of local Romanian safflower germplasm.

Representative HPLC-DAD chromatograms recorded for the evaluated safflower petal extracts and the standard mixture are illustrated in Figure 3a,b. The chromatographic profiles monitoring the extracts at diagnostic wavelengths (280, 320, and 360 nm) revealed a highly dense phytochemical fingerprint characterized by a multitude of peaks. Beyond the major quantified target analytes (such as caffeic and chlorogenic acids), the presence of numerous unassigned chromatographic signals underscores the rich diversity of secondary metabolites within the floral tissue. This complex molecular distribution reflects the intense biosynthetic activity occurring during capitulum ontogeny, further emphasizing the potential of local germplasm as a source of multi-component bioactive ingredients.

### 3.4. Quantitative Analysis of Fat-Soluble Vitamins in Safflower Petals

To ensure analytical prudence, the evaluation of individual phytochemical compounds and fat-soluble vitamins is presented as a preliminary semi-quantitative and comparative screening tailored to characterize these specific gene-bank composite batches, rather than an absolute quantification. The quantification of fat-soluble vitamins within horticultural matrices is essential for evaluating their overall functional and nutraceutical value. In this study, a comparative extraction approach was implemented using a polar solvent (100% ethanol) versus a non-polar solvent mixture (hexane: ethyl acetate, 4:1 *v*/*v*) assisted by ultrasound-assisted extraction (UAE). The concentration of vitamins E (α-tocopherol), and K_1_ (phylloquinone), expressed as milligrams per 100 g of dry weight (mg 100 g^−1^ d.w.), are summarized in Table 4.

The comparative analysis revealed distinct accumulation patterns among the tested germplasm, heavily influenced by both genetic variation and the chemical nature of the extraction solvents. Notably, vitamin K_1_ (phylloquinone) remained below the detection limit across all populations and solvent systems. This absence is biologically normal, as vitamin K1 is produced primarily in green leaves and stems to support photosynthesis, while remaining naturally below detection limits in non-photosynthetic tissues like flower petals. Consequently, this specific metabolite does not accumulate in quantifiable amounts within the petal matrix of these local lines. A key biochemical finding was that 100% ethanol consistently provided a highly efficient extraction recovery for vitamin E (α-tocopherol) representing a notable advantage compared to the traditional non-polar hexane: ethyl acetate mixture. From the perspective of green chemistry this represents a notable advantage, demonstrating that eco-friendly, food-grade, and biologically safe solvents (such as ethanol) can efficiently recover lipophilic fractions without requiring hazardous volatile organic solvents. Across all analytical variations, the investigated spineless lines (var. *inermis*: R3-TR and R4-CL) displayed higher vitamin accumulations than the evaluated spiny genotypes (var. *typicus*: R1-BT and R2-BC). The Călărași population (R4-CL) recorded the maximum levels for vitamin E (8.45 ± 0.19 mg 100 g^−1^ d.w.) under pure ethanol extraction conditions. Nevertheless, these values reflect an initial baseline screening specific to the 2024 cultivation cycle, emphasizing the need for wider multi-environmental trials to properly map the lipophilic stability of these localized landraces.

### 3.5. Yield of Total Natural Dye Content

Safflower petals are commercially valued as an abundant source of non-toxic natural dyes, composed primarily of water-soluble yellow quinochalcones (safflower yellow) and the water-insoluble red pigment known as carthamin. The gravimetric evaluation of the total dye content obtained via acid hydrolysis across the four local Romanian landraces is presented in Table 5.

The experimental results demonstrated a competitive pigment yield for both morphological varieties, with values ranging from a minimum of 4.38% in the spineless population from Teleorman (R3-TR) to a maximum of 5.56% in the spineless population from Călărași (R4-CL). Within the spiny lines, the dye content fluctuated within a narrow range, between 5.54% for R1-BT and 5.20% for R2-BC. A comparative evaluation of these data leads to an important agronomic conclusion: the highest dye concentrations were recorded for the spineless line R4-CL (5.56%) and the spiny line R1-BT (5.54%). In conventional agriculture, spiny genotypes are frequently associated with superior dye quality. However, these preliminary results demonstrate that these specific local spineless lines can match or even exceed the pigment production of the evaluated spiny genotypes under the tested regional conditions. Given that spineless safflower significantly facilitates both manual and mechanical harvesting operations, the competitive dye yield of the R4-CL landrace suggests a promising direction for potentially efficient cultivation with reduced labor requirements, which warrants further validation across multiple years and environments to fully confirm its commercial adaptability for the natural dye industry.

### 3.6. Statistical Analysis and Correlation of Phytochemical Components

One-way Analysis of Variance (one-way ANOVA), complemented by Tukey’s HSD post-hoc test (*p* < 0.05), revealed statistically significant variations among the four local safflower landraces for most of the investigated bioactive parameters. Figure 4 presents the comparative profiling of total phenolic content (TPC), total flavonoid content (TFC), and total natural dye yield across four local Romanian safflower (*Carthamus tinctorius* L., fam. *Asteraceae*) populations. TPC values are expressed as milligrams of gallic acid equivalents per gram of dry weight (mg GAE g^−1^ d.w.); TFC values are expressed as milligrams of quercetin equivalents per gram of dry weight (mg QE g^−1^ d.w.), and total dye yield is expressed as a percentage (%). Different lowercase superscript letters (a–c) placed above the bars indicate statistically significant differences between individual population means for each parameter, determined via one-way ANOVA followed by Tukey’s HSD post-hoc test (*p* < 0.05).

The Pearson correlation matrix revealed several key metabolic relationships within the studied safflower germplasm (Table 6). A very strong positive correlation was observed between TPC and DPPH radical scavenging activity (*r* = −0.92) demonstrating that an increase in phenolic concentration leads to a lower IC_50_ value. This relationship reflects the direct linear dependency of the radical scavenging response on the phenolic concentration within the tested range, confirming that phenolic acids identified via HPLC (such as chlorogenic and caffeic acid) are the primary drivers of antioxidant efficiency [29]. Furthermore, the Total Dye content showed a significant correlation with the ABTS assay (*r* = 0.71), indicating that specific quinochalcone pigments contribute more substantially to the antioxidant capacity measured via ABTS than to the DPPH system. Interestingly, a negative correlation between TFC and Total Dye (*r* = −0.69) was documented, suggesting a potential metabolic competition between the biosynthetic pathways of flavonoids and carthamin-type pigments within these local landraces.

## 4. Discussion

The phenolic composition and antioxidant activity of safflower (*Carthamus tinctorius* L.) petals have been previously investigated [16,24]. These characteristics are modeled by complex interactions between environmental factors and sustainable agricultural practices [2,4,30]. Recent investigations on *Carthamus* species have demonstrated that the distinct accumulation of polyphenolic profiles and specialized metabolic fractions plays a vital functional role, significantly shaping the baseline antioxidant capacity of the tissue [31,32]. Furthermore, the development and application of sustainable, eco-friendly extraction techniques remain crucial for maximizing the recovery yields of these highly specialized bioactive fractions from diverse agricultural matrices [17,33,34]. Previous phytochemical screenings established that gallic acid is a major phenolic acid in *C. tinctorius* florets, followed by chlorogenic and caffeic acids [16,24,29]. These individual compounds play vital biological roles, with well-documented bioactive properties in the literature [24,35,36]. Regarding the petal pigmentation, it is important to clarify that the characteristic yellow coloration of *Carthamus tinctorius* L. florets is primarily driven by quinochalcone glycosides, such as Safflor Yellow A and precarthamin. In this study, the analytical screening via HPLC-DAD was optimized for targeted phenolic acids and flavonoids, monitored at narrow UV wavelengths of 280 nm, 320 nm, and 360 nm. Due to this high detection selectivity, quinochalcone pigments (which typically exhibit maximum light absorption profiles in the visible spectrum above 400 nm) do not display prominent chromatographic peaks under the given operational parameters. The clean baseline is a consequence of these targeted wavelength channels and sample dilution, ensuring well-resolved peaks without any artificial threshold filtering.

Regarding the individual hydroxycinnamic acids quantified in this study, the observed discrepancy between the prominent visual intensity of the chlorogenic acid peak (ChlA) on the chromatograms and the higher quantitative mass concentrations calculated for caffeic acid in Table 3 is governed by distinct instrumental response factors. At the monitored UV wavelengths (280 and 320 nm), chlorogenic acid exhibits a significantly higher molar absorptivity coefficient compared to caffeic acid, thus generating an intense chromatographic signal even at lower absolute quantities. In agreement with the quantitative data, caffeic acid emerged as the primary compound across all investigated Romanian landraces. The spineless phenotypes (var. *inermis*) exhibited a notable accumulation trend, with line R4-CL reaching a maximum of 289.41 ± 5.85 mg 100 g^−1^ d.w., followed by R3-TR with 260.64 ± 4.11 mg 100 g^−1^ d.w. The selection of caffeic acid as a key target analyte is justified by its critical biological functionality, including its role as a principal phenolic constituent in *C. tinctorius* L. florets strongly supported by earlier screenings [24,35,36]. This indicates that regional adaptation towards spineless phenotypes in Romania preserves the metabolic pathway of this dominant antioxidant fraction.

Similarly, chlorogenic acid was positively identified and quantified in all four lines. In contrast to the caffeic acid trend, the highest concentration was recorded in the spiny population R2-BC (45.71 ± 1.13 mg 100 g^−1^ d.w.), while the spineless lines showed intermediate, statistically homogeneous values (31.98–33.13 mg 100 g^−1^ d.w.). This compound was selected due to its therapeutic relevance in managing metabolic disorders. Its presence in our samples aligns with reference literature confirming chlorogenic acid as a standard component of the *C. tinctorius* floral matrix [16,24,29], reflecting a well-conserved biosynthetic pathway among both var. typicus and var. inermis germplasm.

For *p*-coumaric acid and ferulic acid, quantifiable amounts were systematically detected across all lines. The spiny landrace R2-BC synthesized the highest levels of *p*-coumaric acid (57.70 ± 1.54 mg 100 g^−1^ d.w.), whereas ferulic acid remained remarkably stable across all populations, fluctuating within a narrow range of 38.36 to 44.63 mg 100 g^−1^ d.w. without major statistical deviations. These specific hydroxycinnamic derivatives were chosen to evaluate the integrity of the core phenylpropanoid pathway under identical regional field conditions. Their verified presence consolidates previous phytochemical assertions [16,29] that these baseline organic acids are pre-existent and structural components of safflower floral tissues.

Gallic acid, a fundamental hydroxybenzoic acid selected for its exceptional radical-trapping efficiency, displayed an intriguing accumulation pattern. It was quantified at 7.72 ± 3.47 mg 100 g^−1^ d.w. in the spiny R1-BT and reached a maximum of 14.71 ± 5.12 mg 100 g^−1^ d.w. in the spineless R3-TR. Although a distinct chromatographic peak was visible in the R4-CL profile, matrix interferences prevented a reliable quantification (reported as NQ). Its targeted analysis is supported by its role as a key biomarker in *C. tinctorius* florets [16,24], and our findings confirm its pre-existence in Romanian local populations, in agreement with traditional phytochemical profiles.

A study conducted by Salem et al. [24] demonstrated that polyphenol accumulation varies significantly throughout floral ontogeny, reaching maximum levels at the end of the flowering process. Safflower plants under environmental stress can redirect their biosynthetic pathways, leading to an improvement in the secondary metabolite profile of the florets, though this often occurs at the expense of overall seed yield [37]. Furthermore, the development and application of sustainable, eco-friendly extraction techniques remain crucial for maximizing the recovery yields of these highly specialized bioactive fractions from diverse agricultural matrices [33]. In comparison with reference literature data indicating average total polyphenol contents of approximately 3.5 g GAE/100 g d.w. (equivalent to 35 mg GAE g^−1^ d.w.) under limiting conditions [37], the samples analyzed in the present study exhibited comparable values (23.72–25.26 mg GAE g^−1^ d.w.), confirming a robust biosynthetic stability within the studied local Romanian populations.

Polyphenols can exert a dual biological effect; depending on their internal chemical structure, concentration, and the presence of transition metal ions, they can act as effective antioxidants or transition to a pro-oxidant character [38,39,40]. Results presented by Young Lee et al. [41] suggest that flavonoids from safflower leaves, along with luteolin and quercetin, represent potential sources of natural antioxidants capable of inhibiting lipid peroxidation. Within the flavonoid fraction analyzed in this study, the glycosylated flavonols rutin and isoquercetin represented major components of the polyphenolic matrix across all samples. The spiny landrace R1-BT accumulated the highest concentrations of both rutin (95.47 ± 8.69 mg 100 g^−1^ d.w.) and isoquercetin (72.50 ± 4.61 mg 100 g^−1^ d.w.). These compounds were specifically targeted due to their established capacity to synergistically inhibit lipid peroxidation and mitigate intracellular oxidative stress. The baseline presence of these specific glycosides is well-documented in *C. tinctorius* organs, and our quantitative results confirm their steady pre-existence in the floral tissue, serving as primary chemical drivers behind the total flavonoid content (TFC) observed.

In contrast to the flavonol glycosides, the free flavone apigenin and the flavanone naringenin exhibited different accumulation variations among the studied phenotypes. The local genotype R4-CL achieved the highest concentrations for both apigenin (26.30 ± 0.25 mg 100 g^−1^ d.w.) and naringenin (35.76 ± 0.29 mg 100 g^−1^ d.w.). The selection of these specific structural subclasses is justified by their high chemotaxonomic profiling value and well-documented pharmacological potential in natural product research. Their positive identification confirms their pre-existence in the *C. tinctorius* matrix, while the quantitative variations suggest that morphological variations may correlate with distinct shifts in secondary metabolic pathways branching toward free flavones and flavanones.

Finally, kaempferol followed a similar structural distribution, showing higer quantified values in the spineless lines R3-TR (22.29 ± 0.36 mg 100 g^−1^ d.w.) and R4-CL (20.04 ± 0.30 mg 100 g^−1^ d.w.) compared to the spiny phenotype R1-BT (8.14 ± 0.22 mg 100 g^−1^ d.w.), while dropping below the analytical detection limit in population R2-BC. Kaempferol was included in our screening due to its verified therapeutic functionality and known synergistic interactions with other dietary polyphenols. Its detection in these local Romanian landraces solidifies its pre-existence in the species under regional cultivation scenarios and highlights this compound as a useful metabolic marker for evaluating var. *inermis* germplasm profiles.

Regarding the lipophilic micronutrient profile detailed in Table 4, the separate evaluation of fat-soluble vitamins reveals distinct metabolic and analytical patterns influenced by both genetic variation and solvent polarity.

Vitamin E (α-tocopherol) was systematically quantified across all investigated lines, serving as a vital lipophilic antioxidant that stabilizes membrane structures and protects hydrophobic cellular fractions from oxidative degradation. The selection of α-tocopherol is justified by its prominent role in enhancing the shelf-life and nutritional value of plant-based products. The quantitative trends mirror the polyphenolic data, where the spineless genotype R4-CL achieved the highest concentration (8.45 ± 0.19 mg 100 g^−1^ d.w. using 100% ethanol), compared to the spiny landraces R1-BT (5.85 ± 1.11 mg 100 g^−1^ d.w.) and R2-BC (6.33 ± 0.81 mg 100 g^−1^ d.w.). While vitamin E accumulation is extensively documented and confirmed in safflower seeds and cold-pressed seed oils [35,42], our results confirm its pre-existence within the underutilized petal matrix, establishing these floral by-products as an innovative source of dietary tocopherols. Furthermore, the significantly higher recovery using 100% ethanol over the conventional hexane:ethyl acetate mixture (e.g., 8.45 vs. 7.74 mg 100 g^−1^ d.w. for R4-CL) validates the application of green, food-grade solvent systems in horticultural processing [42]. Although lipophilic vitamins generally exhibit higher solubility in non-polar solvent systems, extraction efficiency depends heavily on core matrix characteristics and solute–matrix interactions. Absolute ethanol may have enhanced tissue penetration and promoted the disruption of cellular structures, thereby facilitating the release of intracellular compounds that were less accessible to the hexane: ethyl acetate mixture. Additionally, distinct matrix effects, including potential interactions with proteins, lipids, and cell wall components, may have influenced the final comparative extraction yield, outlining a hypothetical mechanism that requires further targeted investigation to be fully elucidated. Recent data focusing on the recovery of bioactive compounds from agricultural residues confirm that the optimization of green extraction protocols provides a viable option for isolating valuable antioxidant fractions while supporting circular economy frameworks [34].

Notably, vitamin K1 (phylloquinone) remained below the detection limit across all populations and solvent systems. This absence is biologically normal, as vitamin K1 is produced primarily in green leaves and stems to support photosynthesis, while remaining naturally below detection limits in non-photosynthetic tissues like flower petals. Consequently, this specific metabolite does not accumulate in quantifiable amounts within the petal matrix of these local lines. This distinct compartmentalization provides valuable chemotaxonomic baseline data for future crop breeding programs. Nevertheless, these quantitative trends reflect an initial baseline specific to the 2024 growing season at a single location, highlighting the strict necessity for multi-year and multi-environmental evaluations to definitively confirm the climatic stability and accumulation patterns of these localized lipophilic fractions.

In our study, the free radical scavenging activity was evaluated using DPPH, ABTS, and FRAP assays, with phenolic compounds being recognized as primary predictors of antioxidant capacity [20,21,43]. Strong positive correlations were documented between TPC and antioxidant activity (*r* = 0.92), confirming findings by Cafaro et al. [28] and Özkan et al. [44]. The TPC values quantified in the investigated Romanian landraces were higher than those reported by Baydar & Özkan [45] for Turkish cultivars (9.06–16.62 mg GAE g^−1^ d.w.), indicating a robust phytochemical profile for the local germplasm.

Regarding the spectrophotometric evaluation of the Total Dye Content (TDC) presented in Table 5, this parameter was selected as a primary quality indicator due to the historical and industrial prominence of *C. tinctorius* L. as a premium source of natural, non-toxic colorants. Our quantitative results for these Romanian landraces align perfectly with recent regional data on natural pigment extraction from this species [46]. Safflower florets are universally recognized for accumulating two distinct pigment classes: the water-soluble safflower yellows and the water-insoluble quinochalcone carthamin, which provides the characteristic red hue. The targeted analysis of these pigments is highly justified by their complex biochemical behavior; the transition from a yellow to a red hue constitutes direct evidence of carthamin formation from its precursor, precarthamin, a process mediated by the enzyme *β*-glucosidase during flower maturation [47,48]. Additionally, carthamin (red) exhibits lower thermal stability compared to yellow quinochalcones under high diurnal temperatures [49]. Our findings highlight the highly competitive dye yield of the spineless landrace R4-CL (5.56%), confirming that the genetic transition to var. *inermis* does not negatively suppress the secondary metabolic pathways responsible for pigment biosyntheses. The verified pre-existence of these rich pigment profiles in local germplasm, combined with the clear advantages of spineless phenotypes for mechanical harvesting, highlights the potential interest of these lines for sustainable agricultural applications [17,50]. However, it must be noted that this study measured only the total dye yield. Individual pigment fractions, such as carthamin, were not quantified because pure reference standards were not available during this phase. Additionally, the present screening was executed using external standard calibrations without the integration of an internal standard, establishing a comparative baseline for extraction yields rather than a fully verified absolute recovery profile, which remains a technical milestone for subsequent regulatory validation phases. This lack of individual profiling represents a limitation of the current work, and quantifying specific carthamin and safflomin molecules will be priority for our future research.

To explore the functional relationships within the petal matrix, a Pearson correlation analysis was conducted (Table 6). The statistical trends reveal positive correlations between the total phenolic content (TPC), total flavonoid content (TFC), and the individual antioxidant parameters evaluated via DPPH, ABTS, and FRAP assays (*p* < 0.05; *p* < 0.01). Additionally, caffeic acid and rutin displayed high correlation coefficients with the FRAP and ABTS values, suggesting that these specific compounds represent important chemical drivers behind the radical-scavenging activity of the tested extracts. Furthermore, a positive correlation was observed between the total dye content and the hydrophilic antioxidant capacity (*p* < 0.05), indicating that safflower pigments may complement the phenolic fractions in mitigating oxidative stress under the evaluated conditions. This statistical overview highlights that the observed antioxidant response reflects a cumulative contribution of the overall phytochemical profile rather than the action of a single compound. This trend aligns with previous evaluations of safflower petal compositions, where higher contents of total phenols, flavonoids, and natural colorants are generally associated with increased radical-scavenging efficiency of the floral tissue [17]. However, given that this exploratory screening is restricted to four local genotypes, these Pearson correlation outcomes must be interpreted with caution. While they describe internal trends and solute-solvent interactions within this specific experimental matrix, they reflect chemotypic dynamics under specific processing conditions rather than definitive general biological traits across the broader *Carthamus tinctorius* L. species.

## 5. Conclusions

This study compared local Romanian spiny and spineless safflower (*Carthamus tinctorius* L.) genotypes. Our primary analysis shows that these local populations have notable concentrations of polyphenols, flavonoids, and vitamin fractions under the conditions of the 2024 growing season. The preliminary results indicate that the investigated spineless phenotypes (var. *inermis*) are comparable to traditional spiny cultivars (var. *typicus*). Specifically, the spineless line R4-CL exhibited the highest recorded values for total phenolics, natural dye content (5.56%), and vitamin E. Given that spineless safflower significantly facilitates harvesting and processing operations, this specific genotype represents a potential candidate for subsequent evaluation regarding automated agricultural practices tailored for the food, cosmetic, and natural dye industries. Furthermore, the application of 100% pure ethanol demonstrated competitive efficiency as a sustainable, eco-friendly extraction solvent system.

In conclusion, while this screening indicates substantial concentrations of polyphenolic compounds and notable phytochemical traits, these baseline data cannot yet be utilized to definitively confirm wide-scale climate adaptation or direct industrial application, identifying multi-year and multi-location validation as an essential prerequisite for future research. Additional analytical characterizations are required to fully elucidate the individual pigment fractions before any specific commercial models can be proposed, established, or integrated into future breeding programs.

## Figures and Tables

**Figure 2:**
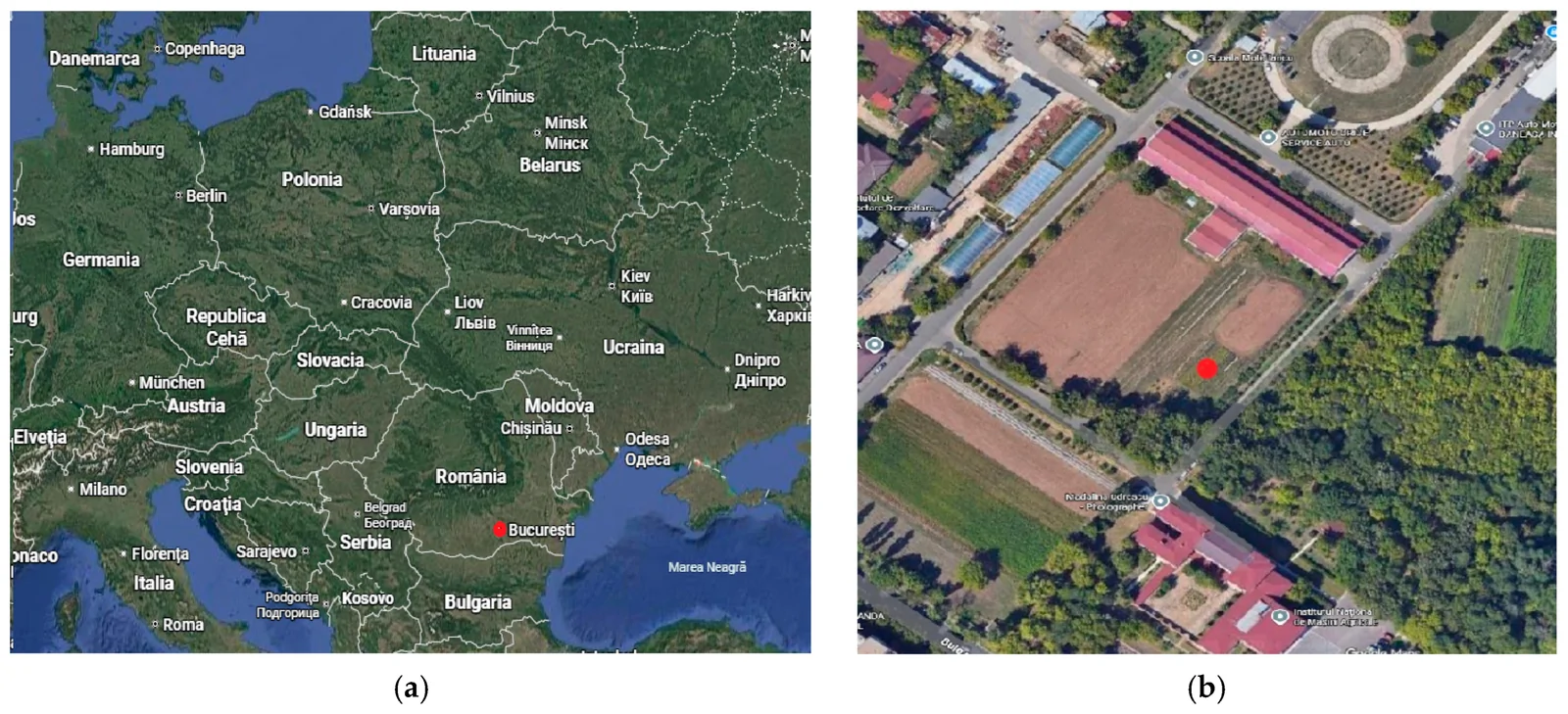
Geographical location and satellite layout of the research site. (**a**) Regional and national geographic context highlighting the location of Bucharest (red circle) within South-Eastern Europe; (**b**) High-resolution satellite layout of the experimental field plots at INMA Bucharest (Banes) utilized for the *Cantharus tinctorial* L. cultivation and phenotypic plasticity trials. The red circle in (**b**) marks the exact location of the ‘experimental safflower plots’ at INMA Bucharest.

**Figure 3:**
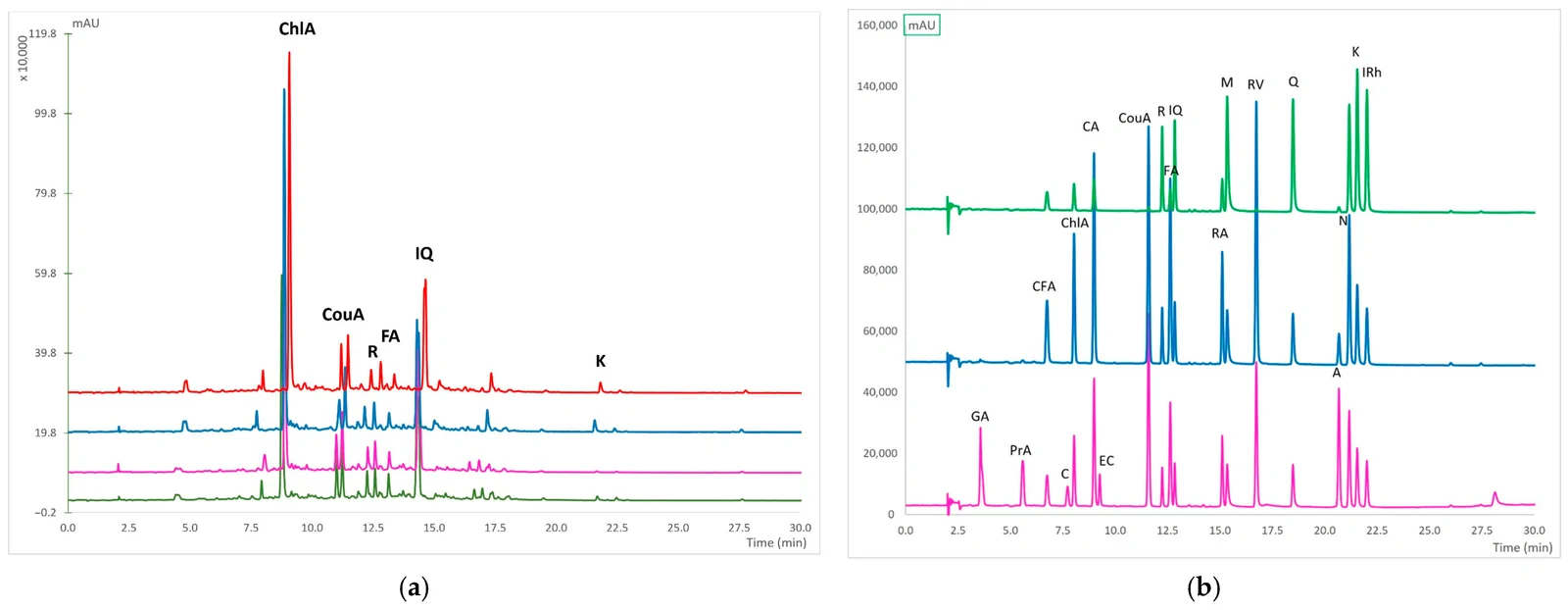
(**a**) Representative HPLC-DAD chromatographic profiles of the polyphenolic fraction from Romanian safflower petals monitored at 280 nm, showing the overlay of the four investigated populations: R1-BT (▬), R2-BC (▬), R3-TR (▬), and R4-CL (▬). (**b**) Chromatograms of standards: (▬) rutin (R), isoquercitrin (IQ), myricetin (M), quercetin (Q), kaempferol (K), and isorhamnetin (IRh) monitored at 360 nm; (▬) caffeic acid (CFA), chlorogenic acid (ChlA), *p*-coumaric acid (CouA), ferulic acid (FA), rosmarinic acid (RA), and naringenin (N) monitored at 320 nm; (▬) gallic acid (GA), protocatechuic acid (PrA), catechin (C), and apigenin (A) monitored at 280 nm.

**Figure 4:**
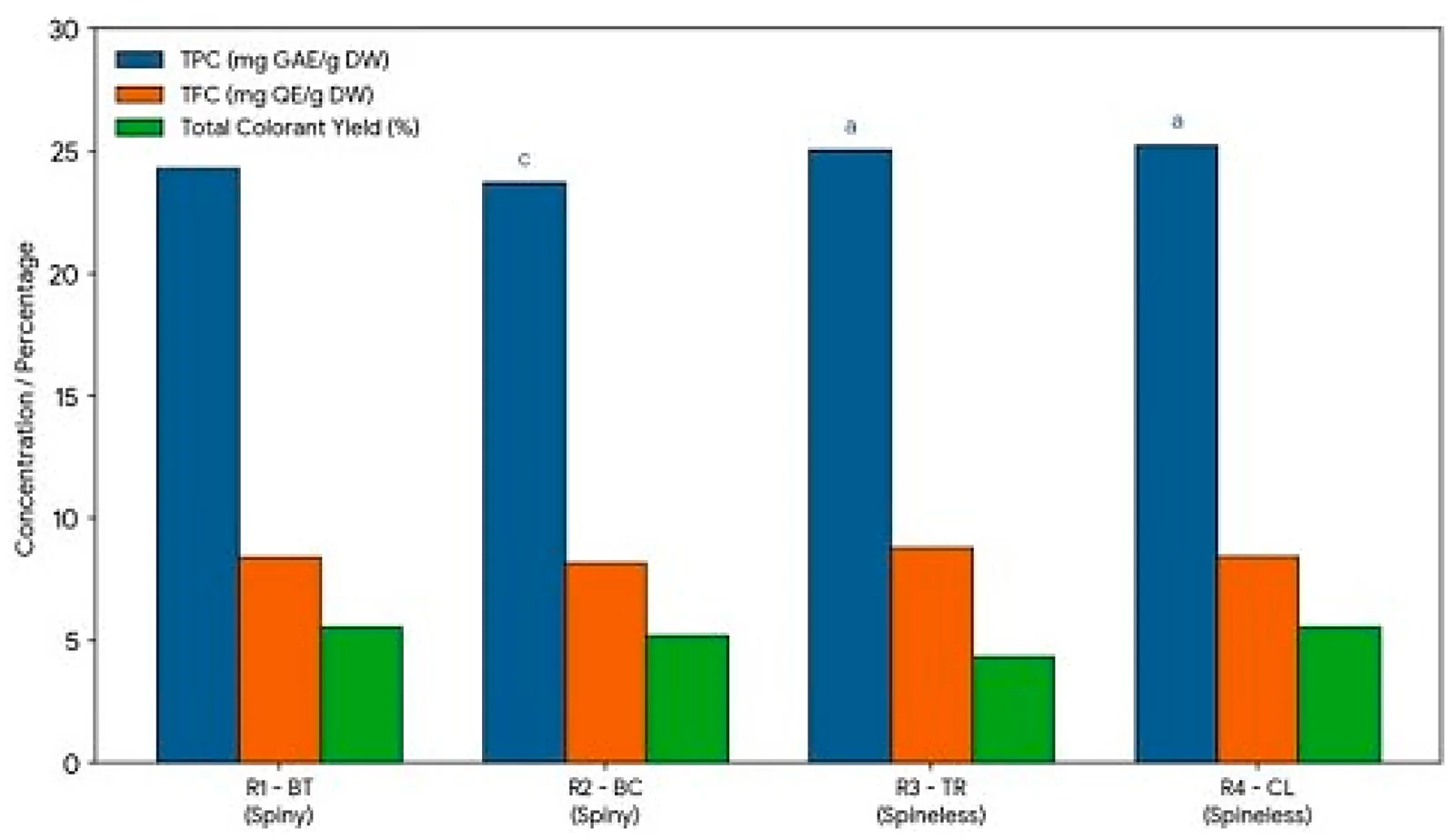
Comparative distribution of total phenolic content (TPC), total flavonoid content (TFC), and total natural dye yield in the studied Romanian safflower landraces. Different lowercase letters (a, c) placed above the bars indicate statistically significant differences between population means for each parameter, determined via one-way ANOVA followed by Tukey’s HSD test (*p* ≤ 0.05).

**Table 1:** Total phenolic content (TPC) and total flavonoid content (TFC) in the experimental safflower petal populations.

Sample	Morphological Variety	TPC (mg GAE g^−1^ d.w.)	TFC (mg QE g^−1^ d.w.)
R1-BT	var. *typicus* (Spiny)	24.33 ± 0.24 ^b^	8.41 ± 0.61
R2-BC	var. *typicus* (Spiny)	23.72 ± 0.13 ^c^	8.18 ± 0.13
R3-TR	var. *inermis* (Spineless)	25.05 ± 0.49 ^a^	8.81 ± 0.26
R4-CL	var. *inermis* (Spineless)	25.26 ± 0.29 ^a^	8.47 ± 0.36

Values represent the mean of four replicates (*n* = 4) ± standard deviation (SD). Different lowercase superscript letters (^a–c^) within the same column indicate statistically significant differences between sample means according to the ANOVA and Tukey’s post-hoc test (*p* < 0.05). d.w., dry weight; GAE, gallic acid equivalents; QE, quercetin equivalents.

**Table 2:** Antioxidant capacity parameters of the experimental safflower petal populations.

Sample	DPPH IC_50_ (µg mL^−1^)	ABTS (mM Trolox g^−1^)	FRAP (mM FeSO_4_ g^−1^)	FRAP (mM Trolox g^−1^)
R1-BT	12.45 ± 0.01 ^b^	140.14 ± 4.06 ^ab^	116.72 ± 1.12 ^a^	121.36 ± 1.31 ^a^
R2-BC	12.09 ± 0.12 ^b^	138.51 ± 2.33 ^b^	113.28 ± 5.06 ^a^	119.19 ± 3.08 ^a^
R3-TR	14.70 ± 0.28 ^a^	137.28 ± 2.82 ^b^	115.55 ± 3.02 ^a^	118.01 ± 2.47 ^a^
R4-CL	14.12 ± 0.00 ^a^	145.71 ± 2.21 ^a^	116.30 ± 3.92 ^a^	121.10 ± 1.76 ^a^

Values represent the mean ± standard deviation (SD) of three independent analytical replicates derived from a single composite average sample per genotype (*n* = 3). Values within the same column sharing different superscript letters (^a,b^) are significantly different based on Tukey’s HSD test at a strict significance level of *p* < 0.05. A lower IC_50_ value denotes higher radical scavenging efficiency. DPPH: 2,2-diphenyl-1-picrylhydrazyl; ABTS: 2,2′-azino-bis(3-ethylbenzothiazoline-6-sulfonic acid); FRAP: ferric reducing antioxidant power.

**Table 3:** Quantitative determination of individual polyphenolic compounds in safflower petal extracts (mg 100 g^−1^ d.w.).

Compound	R1-BT	R2-BC	R3-TR	R4-CL	Literature Source (Citations)
Gallic acid	7.72 ± 3.47 ^b^	ND	14.71 ± 5.12 ^a^	NQ	[23,24,25]
Protocatechuic acid	ND	ND	ND	ND	[26]
Catechin	ND	ND	ND	ND	[23]
Chlorogenic acid	29.91 ± 1.43 ^c^	45.71 ± 1.13 ^a^	31.98 ± 2.86 ^b,c^	33.13 ± 0.73 ^b^	[23,24]
Caffeic acid	207.50 ± 3.08 ^c^	197.85 ± 6.93 ^c^	260.64 ± 4.11 ^b^	289.41 ± 5.85 ^a^	[5,25]
*p*-Coumaric acid	45.75 ± 1.29 ^c^	57.70 ± 1.54 ^a^	48.29 ± 0.79 ^b^	44.89 ± 0.62 ^c^	[25,27]
Rutin	95.47 ± 8.69 ^a^	79.01 ± 4.48 ^a^	71.37 ± 10.14 ^a^	79.24 ± 12.20 ^a^	[5,23,24,28]
Ferulic acid	41.44 ± 6.27 ^a^	44.63 ± 1.52 ^a^	38.36 ± 6.65 ^a^	40.36 ± 3.14 ^a^	[5,25]
Rosmarinic acid	ND	ND	ND	ND	[23]
Isoquercetin	72.50 ± 4.61 ^a^	53.04 ± 2.62 ^b^	62.99 ± 3.77 ^a,b^	56.59 ± 6.95 ^b^	[6,26]
Myricetin	ND	ND	ND	ND	[5]
Quercetin	2.24 ± 0.63 ^a^	ND	ND	ND	[5,23,24]
Apigenin	10.92 ± 0.53 ^d^	14.63 ± 1.03 ^c^	18.09 ± 0.32 ^b^	26.30 ± 0.25 ^a^	[5,26]
Naringenin	9.07 ± 0.27 ^c^	28.24 ± 1.27 ^b^	22.23 ± 0.19 ^b^	35.76 ± 0.29 ^a^	[5,26]
Kaempferol	8.14 ± 0.22 ^b^	ND	22.29 ± 0.36 ^a^	20.04 ± 0.30 ^a^	[5,6,23,26]
Isorhamnetin	5.19 ± 0.74 ^a^	ND	ND	ND	[6]

Values represent the mean ± standard deviation (SD) of three independent analytical replicates derived from a single composite average sample per genotype (*n* = 3). Values within the same row sharing different superscript letters (^a–d^) are significantly different based on Tukey’s HSD test at a strict significance level of *p* < 0.05. NQ: not quantifiable due to suspected matrix interference or co-elution. ND: not detected; d.w.: dry weight.

**Table 4:** Quantitative determination of fat-soluble vitamins in safflower petal extracts (mg·100 g^−1^ d.w.) as a function of the extraction solvent system.

Compound	Extraction Solvent	R1-BT	R2-BC	R3-TR	R4-CL
Vitamin E	EtOH 100%	5.85 ± 1.11 ^b^	6.33 ± 0.81 ^b^	6.67 ± 1.70 ^b^	8.45 ± 0.19 ^a^
	Hex:EtAc (4:1)	4.34 ± 0.08 ^c^	5.70 ± 0.01 ^b^	6.14 ± 0.11 ^b^	7.74 ± 0.36 ^a^
Vitamin K_1_	EtOH 100%	ND	ND	ND	ND
	Hex:EtAc (4:1)	ND	ND	ND	ND

Values represent the mean ± standard deviation (SD) of three independent analytical replicates derived from a single composite average sample per genotype (*n* = 3). Values for the same vitamin sharing different superscript letters (^a–c^) are significantly different based on Tukey’s HSD test at a strict significance level of *p* < 0.05. EtOH: ethanol; Hex:EtAc: hexane:ethyl acetate mixture; ND: not detected; d.w.: dry weight.

**Table 5:** Yield of total natural dye content in the experimental safflower populations.

Parameter	R1-BT (%)	R2-BC (%)	R3-TR (%)	R4-CL (%)
Total dye content	5.54 ± 0.12 ^a^	5.20 ± 0.18 ^ab^	4.38 ± 0.25 ^b^	5.56 ± 0.09 ^a^

Values represent the mean ± standard deviation (SD) of three independent analytical replicates derived from a single composite average sample per genotype (*n* = 3). Values within the same row sharing different superscript letters (^a,b^) are significantly different based on Tukey’s HSD test at a strict significance level of *p* < 0.05.

**Table 6:** Pearson correlation matrix between bioactive compounds and antioxidant activities.

Parameter	TPC	TFC	DPPH	ABTS	FRAP	Total Dye
TPC	1.00					
TFC	0.77 *	1.00				
DPPH (IC_50_)	−0.92 **	−0.88 **	1.00			
ABTS	0.49	−0.17	0.17	1.00		
FRAP	0.67 *	0.49	0.41	0.46	1.00	
Total Dye	−0.19	−0.69 *	−0.54	0.71 *	0.25	1.00

TPC: total phenolic content; TFC: total flavonoid content; DPPH: 2,2-diphenyl-1-picrylhydrazyl; ABTS: 2,2′-azino-bis (3-ethylbenzothiazoline-6-sulfonic acid); FRAP: ferric reducing antioxidant power. * Correlation is significant at the 0.05 level (*p* < 0.05); ** Correlation is significant at the 0.01 level (*p* < 0.01). Note: Correlations are calculated based on landrace-level composite means (*n* = 4) and must be interpreted strictly as preliminary and exploratory trends, rather than definitive or generalized biological traits.

## Data Availability

The data presented in this study are available on request from the corresponding author. The data are not publicly available due to privacy and ethical restrictions.

## Supplementary Materials

The following supporting information can be downloaded at https://www.mdpi.com/article/10.3390/plants15192959/s1: Table S1: Pedoclimatic parameters of the experimental site (INMA Bucharest-Băneasa) during the 2024 cultivation cycle. Table S2: Analytical performance and partial validation parameters of the HPLC-DAD methods for the separation and quantification of individual polyphenols and fat-soluble vitamins (Parts A–D).

## Abbreviations

The following abbreviations are used in this manuscript:

ABTS	2,2′-azino-bis (3-ethylbenzothiazoline-6-sulfonic acid)
ANOVA	Analysis of variance
DAD	Diode array detector
DPPH	2,2-diphenyl-1-picrylhydrazyl
FRAP	Ferric reducing antioxidant power
GAE	Gallic acid equivalents
HPLC	High-performance liquid chromatography
ND	Not detected
NUS	Neglected and underutilized species
RE	Ruthin equivalents
SD	Standard deviation
TDC	Total dye content
TFC	Total flavonoid content
TPC	Total phenolic content
UV-Vis	Ultraviolet-visible spectroscopy
