# Supplementary material for: Phytochemical Variations in Spiny and Spineless Safflower (Carthamus tinctorius L.) Phenotypes
Source: Plants (Basel). 2026 Sep 28;15(19):2959. doi: 10.3390/plants15192959 (PMC13666712; doi:10.3390/plants15192959)
Supplement: Supplementary file 1 [file plants-15-02959-s001.zip › plants-4415319-supplementary.pdf]

Supplementary Materials: Phytochemical Profiling of Romanian Safflower (*Carthamus tinctorius L.*) Landraces

Table S1. Pedoclimatic parameters of the experimental site (INMA Bucharest-Băneasa) during the 2024 cultivation cycle.

Part A: Baseline Soil Physicochemical Properties (Preluvic Luvisol)

Parameter	Value
Soil pH (H_2O)	6.2 – 6.5 (Slightly acidic)
Organic Matter/Humus (%)	2.85 ± 0.14
Total Nitrogen (N) (%)	0.145 ± 0.02
Available Phosphorus (P, Egner method) (mg kg^{-1})	42.1 ± 3.4
Exchangeable Potassium (K) (mg kg^{-1})	185.3 ± 12.0

Note: Soil physicochemical properties represent cumulative historical and seasonal agrochemical profiling data characteristic of the INMA Bucharest-Băneasa experimental fields.

Part B: Climatological Summary (2024 Growing Season)

Month	Average Monthly Temperature (°C)	Cumulative Precipitation (mm)
**April**	14.2	38.5
**May**	18.5	52.1
**June**	23.4	44.2
**July**	26.1	18.4
**August**	15.8	22.1

Note: Climatological parameters (average temperatures and cumulative rainfall) were retrieved from the official regional database of the National Administration of Meteorology (ANM București-Băneasa) corresponding to the 2024 vegetative cycle.

Table S2. Analytical performance and partial validation parameters of the HPLC-DAD methods for the separation and quantification of individual polyphenols and fat-soluble vitamins (Parts A–D).

Part A: Concentration range, calibration curves, regression coefficients, LOD, and LOQ for individual phenolic acids and flavonoids

No.	Compound	Concentration range (μg/ml)	Calibration curve	Regression coefficient (R^2)	LOD (μg/ml)	LOQ (μg/ml)
1	Gallic acid	1-50	y = 25820x - 1572	0.999	0.24	0.79
2	Protocatechuic acid	1.4 - 35	y = 47684x + 18646	0.999	0.78	1.54
3	Catechin	2.5 -50	y = 7626.5x + 648	0.999	0.17	0.56
4	Chlorogenic acid	1-50	y = 15717x - 3589	0.999	0.21	0.66
5	Caffeic acid	1-50	y = 56622x - 19137	0.999	0.35	0.92
6	*p*-Coumaric acid	0.5 - 45	y = 103538x + 5534	0.999	0.11	0.36
7	Rutin	3.5 – 38.5	y = 10968x + 47387	0.998	0.64	2.13
8	Ferulic acid	0.5 - 45	y = 58190x + 10678	0.999	0.17	0.32
9	Isoquercetin	2.5-50	y = 18331x - 1490	0.999	0.33	1.09
10	Rosmarinic acid	1-50	y = 19342x + 5943	0.999	0.61	1.03
11	Myricetin	1-47.5	y = 54519x - 17384	0.999	0.28	0.93
12	Quercetin	1-50	y = 43132x - 2292	0.999	0.21	0.69
13	Apigenin	2.5 – 50	y = 199337x + 56262	0.996	0.56	1.48
14	Naringenin	1- 50	y = 81722x - 29338	0.994	0.44	0.96
15	Kaempferol	1-50	y = 53953x - 17030	0.999	0.26	0.86
16	Isorhamnetin	1-50	y = 61549x - 26363	0.999	0.41	1.02

Part B: Partial validation parameters and precision data for HPLC-DAD method of polyphenols separation

No.	Compound	Intra-assay Precision RSD% (*n*=5, 5 μg/ml)	Inter-assay Precision[a] RSD% (*n*=5, 10 μg/ml)	Inter-assay Precision RSD% (*n*=5, 20 μg/ml)	Recovery % (*n*=3)
1	Gallic acid	3.04	2.36	4.14	88.3
2	Protocatechuic acid	2.65	2.14	3.26	98.6
3	Catechin	2.65	3.53	2.04	101.6
4	Chlorogenic acid	2.01	2.10	3.17	104.2
5	Caffeic acid	1.66	2.26	2.29	98.6
6	*p*-Coumaric acid	1.85	3.37	4.05	99.6
7	Rutin	2.61	4.73	3.65	87.5
8	Ferulic acid	2.13	3.97	3.42	97.8
9	Isoquercetin	3.04	4.14	3.55	103.8
10	Rosmarinic acid	2.16	3.22	2.68	102.8
11	Myricetin	2.48	2.25	2.87	98.8
12	Quercetin	2.06	3.16	3.61	99.6
13	Apigenin	1.85	3.64	4.03	103.3

14	Naringenin	2.27	2.96	3.08	106.4
15	Kaempferol	2.68	2.58	3.62	98.9
16	Isorhamnetin	3.48	3.12	3.84	105.7

Part C: Calibration curve, regression coefficients, LOD, LOQ, for HPLC-DAD fat-soluble vitamins separation

No.	Compound	Concentration range (µg/ml)	Calibration curve	Regression coefficient (R^2)	LOD (µg/ml)	LOQ (µg/ml)
1	Vitamin E	2.5-50	y = 18005.05x -4948.07	0.9976	0.62	2.06
2	Vitamin K1	3-60	y = 86424.3x + 41652.71	0.9998	2.68	8.84

Part D: Partial validation parameters and precision data for HPLC-DAD method fat-soluble vitamins separation

No.	Compound	Intra-assay Precision RSD% (*n*=5, 10 µg/ml)	Intra-assay Precision RSD% (*n*=5, 25µg/ml)	Inter-assay Precision RSD% (*n*=5, 10 µg/ml)	Inter-assay Precision RSD% (*n*=5, 25µg/ml)	Recovery[1] % (*n*=3)	Recovery[2] % (*n*=3)
1	Vitamin E	2.61	0.41	1.81	0.65	102.5	95.4
2	Vitamin K1	0.50	0.79	0.62	0.83	107.6	93.2

[1] EtOH 100% petals extract diluted five times, spiked with known concentrations of standard solutions (15 µg/ml)

[2] Hexane:Ethyl Acetate (4:1, v/v) petals extract diluted five times, spiked with known concentrations of standard solutions (15 µg/ml)
